# Diagnostic value of the isoenzymatic index of lactate dehydrogenase in blood serum in a special process for training cadet pilots

**DOI:** 10.3389/fphys.2024.1399446

**Published:** 2024-12-06

**Authors:** Zbigniew Wochyński, Ireneusz Majsterek, Joanna Gerszon, Radosław Wojtczak, Jacek Kabziński, Jan Błaszczyk, Krzysztof A. Sobiech, Ewa Jabłońska, Wioletta Ratajczak-Wrona

**Affiliations:** ^1^ Department of Air Transport Safety, Polish Air Force University, Deblin, Poland; ^2^ Department of Clinical Chemistry and Biochemistry, Medical University, Lodz, Poland; ^3^ Kaliska Academy President Stanisław Wojciechowski Akademia Kaliska im. Prezydenta Stanisława Wojciechowskiego, Kalisz, Poland; ^4^ Department of Human Biology, University School of Physical Education, Wrocław, Poland; ^5^ Department of Immunology Medical University of Bialystok, Białystok, Poland

**Keywords:** lactate dehydrogenase, isoenzymatic index, aldolase, haptoglobin, C-reactive protein, physical fitness

## Abstract

**Objectives:**

Special aerial gymnastics instruments (SAGI) are permanent elements of specialist training for cadet pilots. Appropriate physical activity and fitness are essential for performing the tasks of military pilots. Therefore, one of the main goals of cadet training is to develop adaptation to the extreme conditions of a military pilot’s work. This study aimed to determine the effect of the SAGI training process for cadet pilots on their aldolase and lactate dehydrogenase (LDH) activity, LDH enzymatic index (iZO), C-reactive protein (CRP) and haptoglobin (HP) levels, and physical fitness.

**Material and Methods:**

Participating in this study were 55 cadets, aged 20 ± 0.70 years, in two groups. In group A (n = 41, tested) cadets were trained on SAGI with an emphasis on endurance and strength. In group B (n = 14, control), the cadets performed general fitness training. Blood samples were collected before and after training series I, II, and III. LDH and its isoenzyme activity, aldolase, CRP, and HP levels were assayed in blood serum using commercially available tests, and the iZO value was calculated.

**Results:**

A downward trend was observed in HP and CRP levels in both groups after all three training series. In group A after training, LDH and aldolase activity was lower than in group B. However, in both groups, a significant increase of iZO values was observed after training series II and III in group A, *p* < 0.001, *p* < 0.05, respectively, and in group B, *p* < 0.05 and *p* < 0.0005, respectively. Physical fitness also improved in both groups.

**Conclusion:**

The course of the SAGI exercises showed that the iZO value depends on physical exercise intensity, its type, and cadet adaptation.

## 1 Introduction

Special aerial gymnastics instruments (SAGI) are permanent elements of specialist cadet training in the Polish Air Force Academy. These instruments include the looping, gyroscope, and aero wheel. Earlier research has included photos of SAGI (Wochynski and Sobiech, 2014; Wochyński and Sobiech, 2015; Wochyński and Sobiech, 2017). Appropriate physical activity and endurance are essential for performing the tasks of military pilots. Therefore, one of the main goals of cadet training is to develop adaptation to the extreme conditions of a military pilot’s work. Hence, determining the degree of body adaptation during specific training on SAGI is indispensable. This requires the use of valuable diagnostic markers which enable to assess training load. Among the variables, C-reactive protein (CRP) and haptoglobin (HP) are classified as acute phase proteins. Additionally, there is evidence that the concentration of CRP (Pedersen et al., 1997) and HP (Kłapcińska et al., 2001) increases after exercise. Consequently, we investigated the effect of specific and exhausting SAGI exercises on CRP and HP levels. Furthermore, the serum activity of the enzymes which indicate the degree of metabolic adaptation to the physical training of skeletal muscles, such as aldolase (EC 4.1.2.13), lactic dehydrogenase (EC 1.1.1.27) (LDH), and the designed LDH isoenzymatic index (iZO), were determined.

Aldolase is key glycolytic enzyme which catalyzes the breakdown of fructose 1,6-bisphosphate into glyceraldehyde 3-phosphate and dihydroxyacetone phosphate. Due to its role in glycolysis, aldolase is mainly localized in the cytoplasm. There is also nuclear fraction, which is found in the heterochromatin region (Mamczur and Dzugaj, 2008). There are three aldolase isoforms (A, B, and C). Isoform A (muscle type) binds to the actin-containing filament of the cytoskeleton, exhibiting a tissue-specific binding pattern. It has been demonstrated that athletes have a considerably higher level of serum aldolase activity during rest than nonathletes. This effect is a result of higher levels and proportions of aldolase A, which predominates in the muscle (Gimenez and Florentz, 1984). Another investigated protein, lactate dehydrogenase, is a tetrameric, cytoplasmic enzyme which comprises H and/or M subunits. Like aldolase, it also participates in the metabolic pathway, catalyzing the final step in anaerobic glycolysis, which converts pyruvate to lactate and NADH to NAD^+^. There are five LDH isoenzymes: LD1 (H_4_), LD2 (H_3_M), LD3 (H_2_M2), LD4 (HM_3_), and LD5 (M4). An increase in LDH activity was observed in athletes after testing, including intensive (supramaximal) physical exercise (Sobiech et al., 2003). iZO was also used to evaluate labor effort (Karmowski et al., 2005). The activity of both enzymes depended on the duration of exercise rather than its intensity (Karamizrak et al., 1994).

Thus far, there are data about changes in the LDH and aldolase activity in athletes during training, but little is known about the degree of pilots’ adaptation under extreme conditions. Appropriate training on SAGI requires monitoring the pilot’s body load. Therefore, it is important to determine whether low-intensity SAGI exercise (in comparison to the control group) loads the body to achieve appropriate (adequate) levels of physical fitness in cadets in the time needed to execute military tasks.

We hypothesize that training on SAGI will exert an effect on CRP and HP levels and iZO values, showing a shift of isoenzymes related to the character of physical exercise, adaptation, and different intensity in the metabolic aerobic processes in comparison to the control group. We assume that changes in the aldolase and LDH activity induced by exercises on SAGI might be a valuable tool in determining adaptation to effort.

## 2 Material and methods

### 2.1 Study population

A total of 55 cadets, men aged 20 ± 0.70, participated in the study. They were divided into two groups. Group A (n = 41, tested) included cadet pilots trained on SAGI. In group B (n = 14), the cadets were a control group and trained according to a standard physical education program. In both groups, the training programs consisted of 20 training units. The entire educational program lasted 70 days, and each training session lasted 90 min. All participants in the experiment were examined by the medical commission of the Military Institute of Aviation Medicine. Group A was classified in the highest health category, Z-1A, and group B in the health category Z-1C. Group A was aged 20.53 ± 1.02 years and had the following somatic characteristics: body height 176.64.70 ± 5.78 cm; body weight 71.03 ± 6.94 kg; BMI 22.82 ± 1.95 kg‧m^−1^; mean heart rate (HR) from training unit: training I–108,5 ± 11,8 bpm; training II–107,3 ± 11,7 bpm; training III- 101,7 ± 7,69 bpm. Group B was aged 20.21 ± 0.42 years and had the following somatic characteristics: body height 173.62 ± 5.12 cm; body weight 67.59 ± 6.36 kg; BMI 22.49 ± 1.87 kg‧m^−1^. Mean HR (bpm) from training unit: training I- 131.6 ± 15,8 bpm; training II- 139,7 ± 8,8 bpm; training III- 145,3 ± 6,7 bpm. Based on the comparison of the two groups, no intergroup differences were observed for age (*p* = 0.96), body height (*p* = 0.42), body weight (*p* = 0.67), or BMI (*p* = 0.78). In group B, a statistically significant increase in HR was observed at *p* < 0.001 in training series I, II, and III in relation to group A. Training series I, II and III were tested in order to assess the course of training adaptation in the officer cadet training process.

### 2.2 Blood collection

**FIGURE 1:**
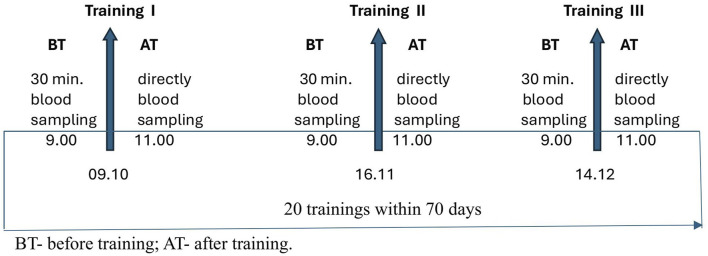
Scheme of the study.

In both groups, the blood samples were collected twice before (BT) and after training (AT); at the beginning (training I), during (training II), and after (training III) completion of the SAGI training program. Blood samples were collected before each training session in the morning at 9:00 am and after training at 11:00 am. Blood samples were collected 30 min before and immediately after training (Figure 1).

### 2.3 Protein and enzyme assays

C-reactive protein (CRP) and haptoglobin (HP) levels were assayed in the blood serum with the immunoturbidimetric technique using reagents by Roche in Integra 400/800 (Germany). The human CRP kit, number CYT 298, and the human Hpt/HP Elisa kit, number 864-EK700066, were used. CRP was expressed in mg/L units and HP in g/L units. Aldolase activity was assayed photometrically by a decrease in absorbance. For each mole of substrate hydrolyzed, 2 mol of coenzyme (NADH) was oxidized. This 1:2 ratio was applied in the calculation of aldolase activity. The human aldolase ALD ELISA kit was used, catalog number: 576-201-12-0838. The aldolase activity was expressed in U/L units. LDH by IFCC technique used reagents by Roche in Integra 400/800 (Germany), and LDH isoenzymes with electrophoresis. The lactate dehydrogenase (LDH) colorimetric activity kit catalog number: EEA013 was used. LDH was expressed in U/L units and LDH isoenzymes in U/I units. Results of LDH isoenzyme assays were used to calculate LDH enzymatic index [8], using the following formula:

iZO=LD4+LD 5LD1+LD2+LD 3.

### 2.4 Training intensity assessment

Training intensity was monitored in both groups using POLAR TEAM-2 PRO by measuring the HR, the changes of which were used to determine the zone of aerobic and anaerobic metabolism during physical effort (training intensity). HR during training was recorded using a recorder on a belt. The belt was placed at chest height during training. After training, the HR recorder was connected to a HR reading device.

### 2.5 Procedure and design

#### 2.5.1 Training program on SAGI

In group A, the SAGI training program was implemented on a looping, gyroscope, and aero-wheel (Wochyński et al., 2010a). The intensity of the exercises in the three training units was similar. The program consisted of different types of exercise.

a) Looping exercises were performed in the sagittal plane forward (ten circles) and backward (ten circles). There was a 20-s break between the circles. The time to change the indicated device was 3 min.
b) Gyroscope exercises were performed in the sagittal plane (circles forward and backward—60 s), frontal (circles left and right—60 s), and longitudinal axis (turn left and right—60 s). Exercises in the gyroscope were performed twice with a break of 20 s. The time to change the indicated device was 3 min.
c) Exercises in the aero-wheel were performed in the frontal (circles left and right—three times) and sagittal plane (circles forward and backward—three times). Exercises in the Rhine wheel were performed in four series with a break of 20 s between them. The time to change the indicated device was 3 min.

Training series I, II, and III were performed as low-intensity endurance and strength exercises. The main goal of the SAGI exercises was to raise the vegetative–vestibular system to a higher level of functioning (spatial orientation, eye–hand coordination, balance, and air sickness). Therefore, a marker of high diagnostic value (iZO) shown in previous studies was used in the study. The authors sought to investigate in detail the effect of SAGI exercises on changes in iZO in cadet pilots during a special training process.

#### 2.5.2 Standard program of physical education

The Standard Physical Education Program is designed to train officer cadets throughout their studies. In group B, the intensity of exercises in all three training series was similar. The physical education program included general improvement exercises with an emphasis on all motor skills necessary for soldiers (training military). Team games, athletics, gymnastics, and strength training were conducted to increase the body’s efficiency and adapt to a greater load at a later stage of education. During the classes, the task, repetitive, and interval methods were used.

In groups A and B, in addition to the training program, the cadets additionally performed all-day physical activity, including exercises, marches, and other military activities during the experiment. The physical fitness of the cadets was assessed before (series I) and after (series II) the training process using fitness tests such as the aviation synthetic efficiency test (ASET) (Wochyński et al., 2010a; Wochyński et al., 2010b; Wochyński et al., 2020; Wochyński et al., 2021; Wochyński, 2022; Prokopczyk and Wochyński, 2022; Tomczak, 2024), 10 × 10 m pendulum run, pull-ups, 16.5 m distance run, and sit-ups. All examined cadets were provided with the same food and accommodation, with a diet in accordance with mass catering standards. The daily ration contained 4,500 kcal, including 150 g fat (30%), 112.5 g protein (10%), and 675 g carbohydrates (60%).

### 2.6 Statistical analysis

The average and standard deviations of all variables were calculated in groups A and B in the statistical study. The Kolmogorov–Smirnov test examined the normal distribution of variables. The assumption regarding the homogeneity of variance was verified by Levene’s test. The difference between the indicators before and after training in both groups was calculated using two-way analysis of variance (ANOVA) with repeated measures for dependent groups and Tukey’s honest significant difference (HSD) test for pairwise *post hoc* comparisons. The effect size for dependent groups before and after training was calculated using Cohen’s D-test and for the independent groups with the Hedges’ g test (Cohen, 1988). The effect sizes were calculated according to the following criteria: small (d = 0.20–0.49), moderate (d = 0.50–0.79), and large (d > 0.80). The G * Power program was used to assess the sample size (Faul et al., 2007). To evaluate the sample size with the size effect f^2^ = 0.35, we assumed an alpha error of 0.05 and a test power of 0.80. The required size of the total sample was estimated at 55 people. Statistical analysis of the test results was performed using STATISTICA 9 statistical software. Differences in average values were considered significant when the calculated *p*-value was less than 0.05.

## 3 Results

The results obtained indicated that in both groups, changes in CRP and HP levels BT and AT I, II, and III were insignificant (Tables 1, 2). The comparison shows that the changes in CRP and HP concentrations in both groups were within the reference ranges (CRP <5 mg/L and HP < 3.3 g/L). In groups A and B, no significant changes in aldolase activity were found after training sessions I, II, and III compared to BT values (see Tables 1, 2). A statistically significant difference was found between groups A and B in aldolase concentration before and after training session I (see Tables 3, 4).

**TABLE 1:** Changes in the acute phase protein levels and enzymatic indices before and after exercises on special aviation gymnastic instruments (SAGI) during training series I, II, and III in group A (N = 41, tested).

Parameter	Training I					Training II					Training III				
	BT	AT	Cohen’s d test	F	p	BT	AT	Cohen’s d test	F	p	BT	AT	Cohen’s d test	F	p
CRP (mg/L)	1.24 ±0.89	1.17 ±0.81	0.08	0.00	0.99	1.12 ±0.86	1.15 ±1.00	0.03	0.00	0.99	0.48 ±0.20	0.49 ±0.21	0.04	0.009	0.92
HP (g./L)	1.21 ±0.61	1.11 ±0.53	0.17	0.52	0.47	1.03 ±0.50	1.02 ±0.53	0.01	1.03	0.31	0.81 ±0.41	0.77 ±0.41	0.09	0.17	0.67
LDH (U/I)	358.1 ±82.1	362.2 ±69.2	0.05	0.05	0.80	326.5 ±48.3	339.2 ±48.2	0.26	1.58	0.21	317.0 ±42.2	313 ±42.5	0.09	0.17	0.67
LD -1 (U/I)	64.2 ±11.2	62.1 ±8.3	0.21	0.90	0.34	62.8 ±16.2	57.4 ±18.1	0.31	1.39	0.24	68.2 ±11.0	63.1 ±10.2	0.48	5.43	<0.05
LD – 2 (U/I)	106.3 ±14.2	99.1 ±13.0	0.52	0.15	0.69	96.3 ±17.1	89.3 ±17.2	0.40	3.87	<0.05	99.3 ±15.4	93.2 ±13.4	0.42	4.04	<0.05
LD – 3 (U/I)	70.2 ±11.1	66.3 ±10.2	0.36	3.16	0.07	72.1 ±14.3	79.0 ±14.2	0.48	4.58	<0.05	64.3 ±11.4	65.1 ±10.3	0.07	0.20	0.65
LD – 4 (U/I)	42.4 ±13.2	44.3 ±11.1	0.15	0.51	0.47	40.0 ±9.4	47.1 ±10.2	0.72	12.44	<0.001	34.2 ±8.1	37.4 ±8.3	0.39	2.49	0.11
LD – 5 (U/I)	79.1 ±56.3	89.0 ±50.3	0.18	0.66	0.41	54.3 ±18.1	65.4 ±14.2	0.68	9.01	<0.005	49.1 ±14.0	53.2 ±18.1	0.25	1.48	0.22
iZO	0.52 ±0.27	0.59 ±0.26	0.26	1.01	0.31	0.41 ±0.12	0.51 ±0.11	0.86	12.85	<0.001	0.36 ±0.09	0.41 ±0.11	0.49	4.72	<0.05
Aldolase (U/L)	7.33 ±3.77	8.10 ±3.70	0.20	2.70	0.10	5.55 ±1.70	5.74 ±1.54	0.11	1.81	0.18	5.75 ±1.55	5.50 ±1.23	0.17	0.60	0.43

BT, before training; AT, after training; p-level of significance; mean ± standard deviation.

**TABLE 2:** Changes in acute phase protein levels and enzymatic indices before and after training series I, II, and III in group B (N = 14, control).

Parameter	Training I					Training II					Training III				
	BT	AT	Cohen’s d test	F	p	BT	AT	Cohen’s d test	F	p	BT	AT	Cohen’s d test	F	p
CRP (mg/L)	1.40 ±0.67	1.37 ±0.58	0.04	0.006	0.93	1.22 ±0.63	1.29 ±0,87	0.0009	0.0005	0.98	0.60 ±0,45	0.60 ±0,44	0.00	0.00	1.00
HP (g./L)	0.99 ±0.44	0.87 ±0.44	0.27	0.47	0.49	0.93 ±0.37	0.85 ±0.40	0.20	0.31	0.58	0.96 ±0.43	0.83 ±0.44	0.29	0.56	0.45
LDH (U/I)	394.3 ±68.4	471.1 ±87.2	0.98	6.76	<0.05	321.1 ±49.1	378.2 ±59.3	1.04	7.50	<0.05	321.3 ±59.1	371.2 ±69.4	0.77	4.23	<0.05
LD -1 (U/I)	84.2 ±9.4	93.4 ±8.2	1.04	7.66	<0.05	63.3 ±7.0	67.2 ±6.1	0.59	1.61	0.21	70.2 ±10.3	71.2 ±14.6	0.07	0.07	0.78
LD – 2 (U/I)	129.3 ±17.4	143.1 ±18.3	0.77	3.97	0.05	99.2 ±15.1	108.1 ±20.3	0.49	1.54	0.22	98.3 ±19.2	102.2 ±18.1	0.20	0.27	0.60
LD – 3 (U/I)	77.2 ±13.1	85.4 ±11.2	0.67	4.69	<0.05	70.0 ±13.2	77.1 ±17.4	0.45	1.33	0.25	66.1 ±11.1	73.0 ±12.1	0.59	2.31	0.14
LD – 4 (U/I)	40.0 ±12.4	54.1 ±15.3	1.01	6.73	<0.05	37.0 ±9.3	49.2 ±9.4	1.30	11.46	<0.005	36.1 ±8.0	50.2 ±9.3	1.62	19.18	<0.0005
LD – 5 (U/I)	62.1 ±40.5	92.0 ±75.0	0.49	1.75	0.19	49.2 ±22.6	76.0 ±22.2	1.19	9.71	<0.005	49.0 ±15.2	73.1 ±24.4	1.18	9.05	<0.01
iZO	0.35 ±0.17	0.46 ±0.32	0.004	1,23	0.27	0.37 ±0.14	0.50 ±0.12	0.99	6.23	<0.05	0.36 ±0.07	0.50 ±0.10	1.62	16.58	<0.0005
Aldolase (U/L)	7.37 ±5.24	9.96 ±4.95	0.50	4.01	0.05	5.46 ±1.26	5.98 ±1.41	0.38	2.10	0.15	5.24 ±1.25	5.97 ±1.34	0.56	2.19	0.15

BT, before training; AT–after training; p-level of significance; mean ± standard deviation.

**TABLE 3:** Changes in LDH concentration and isoenzymatic indicators, aldolase, and acute phase proteins before training series I, II and III in groups A (N = 41, tested) and B (N = 14, control) and comparison between groups. p-level of significance; mean ± standard deviation.

Parameter	Training I					Training II					Training III				
	Group A	Group B	Hedges g test	F	p	Group A	Group B	Hedges g test	F	p	Group A	Group B	Hedges g test	F	p
CRP (mg/L)	1.24 ±0.89	1.40 ±0.67	0.19	0.33	0.56	1.12 ±0.86	1.22 ±0.63	0.12	0.33	0.56	0.48 ±0.20	0.60 ±0.45	0.42	1.79	0.18
HP (g./L)	1.21 ±0.61	0.99 ±0.44	0.38	1.44	0.23	1.03 ±0.50	0.93 ±0.37	0.21	0.54	0.46	0.81 ±0.41	0.96 ±0.43	0.36	1.24	0.26
LDH (U/I)	358.1 ±82.1	394.3 ±68.4	0.48	2.14	0.14	326.5 ±48.3	321.1 ±49.1	0.11	0.08	0.76	317.0 ±42.2	321.3 ±59.1	0.09	0.08	0.77
LD -1 (U/I)	64.2 ±11.2	84.2 ±9.4	1.85	33.21	<0.0001	62.8 ±16.2	63.3 ±7.0	0.01	0.08	0.76	68.2 ±11.0	70.2 ±10.3	0.18	0.21	0.64
LD – 2 (U/I)	106.3 ±14.2	129.3 ±17.4	1.52	36.23	<0.0001	96.3 ±17.1	99.2 ±15.1	0.17	0.28	0.59	99.3 ±15.4	98.3 ±19.2	0.06	0.05	0.80
LD – 3 (U/I)	70.2 ±11.1	77.2 ±13.1	0.60	3.46	0.06	72.1 ±14.3	70.0 ±13.2	0.14	0.15	0.69	64.3 ±11.4	66.1 ±11.1	0.15	0.21	0.64
LD – 4 (U/I)	42.4 ±13.2	40.0 ±12.4	0.18	0.22	0.63	40.0 ±9.4	37.0 ±9.3	0.31	0.65	0.42	34.2 ±8.1	36.1 ±8.0	0.23	0.38	0.53
LD – 5 (U/I)	79.1 ±56.3	62.1 ±40.5	0.32	1.17	0.28	54.3 ±18.1	49.2 ±22.6	0.26	0.66	0.41	49.1 ±14.0	49.0 ±15.2	0.006	0.01	0.89
iZO	0.52 ±0.27	0.35 ±0.17	0.68	4.72	<0.05	0.41 ±0.12	0.37 ±0.14	0.31	0.88	0.35	0.36 ±0.09	0.36 ±0.07	0.00	0.0002	0.98
Aldolase (U/L)	7.33 ±3.77	7.37 ±5.24	0.009	7.53	<0.01	5.55 ±1.70	5.46 ±1.26	0.05	0.05	0.81	5.75 ±1.55	5.24 ±1.25	0.34	1.22	0.27

p-level of significance; mean ± standard deviation.

**TABLE 4:** Changes in LDH concentration and isoenzymatic indicators, aldolase, and acute phase proteins after training series I, II, and III in groups A (N = 41, tested) and B (N = 14, control) and comparison between groups.

Parameter	Training I					Training II					Training III				
	Group A	Group B	Hedges g test	F	p	Group A	Group B	Hedges g test	F	p	Group A	Group B	Hedges g test	F	p
CRP (mg/L)	1.17 ±0.81	1.37 ±0.58	0.26	0.33	0.56	1.15 ±1.00	1.29 ±0.87	0.14	0.33	56	0.49 ±0.21	0.60 ±0.44	0.38	1.96	0.16
HP (g./L)	1.11 ±0.53	0.87 ±0.44	0.47	2.26	0.13	1.02 ±0.53	0.85 ±0.40	0.33	0.69	0.40	0.77 ±0.41	0.83 ±0.44	0.14	0.19	0.66
LDH (U/I)	362.2 ±69.2	471.1 ±87.2	1.47	22.62	<0.00005	339.2 ±48.2	378.2 ±59.3	0.76	5.87	<0.02	313 ±42.5	371.2 ±69.4	1.15	13.94	<0.0005
LD -1 (U/I)	62.1 ±8.3	93.4 ±8.2	3.78	151.08	<0.000001	57.4 ±18.1	67.2 ±6.1	0.61	3.36	0.07	63.1 ±10.2	71.2 ±14.6	0.70	5.64	<0.05
LD – 2 (U/I)	99.1 ±13.0	143.1 ±18.3	3.03	89.29	<0.000001	89.3 ±17.2	108.1 ±20.3	1.04	11.32	<0.002	93.2 ±13.4	102.2 ±18.1	0.61	3.79	0.05
LD – 3 (U/I)	66.3 ±10.2	85.4 ±11.2	1.82	44.11	<0.000001	79.0 ±14.2	77.1 ±17.4	0.12	0.13	0.71	65.1 ±10.3	73.0 ±12.1	0.73	4.68	<0.05
LD – 4 (U/I)	44.3 ±11.1	54.1* ±15.3	0.79	6.34	<0.02	47.1 ±10.2	49.2 ±9.4	0.20	0.31	0.57	37.4 ±8.3	50.2 ±9.3	1.49	25.35	<0.00001
LD – 5 (U/I)	89.0 ±50.3	92.0 ±75.0	0.05	0.02	0.86	65.4 ±14.2	76.0 ±22.2	0.64	3.85	0.05	53.2 ±18.1	73.1† ±24.4	1.00	9.83	<0.005
iZO	0.59 ±0.26	0.46 ±0.32	0.47	1.92	0.17	0.51 ±0.11	0.50 ±0.12	0.08	0.03	0.84	0.41 ±0.11	0.50 ±0.10	0.83	6.35	<0.02
Aldolase (U/L)	8.10 ±3.70	9.96 ±4.95	0.46	9.86	<0.005	5.74 ±1.54	5.98 ±1.41	0.15	0.98	0.32	5.50 ±1.23	5.97 ±1.34	0.37	1.40	0.24

p-level of significance; mean ± standard deviation.

There were no statistically significant differences between groups A and B in the parameters CRP and HP after all three training sessions. (see Tables 3, 4).

In group A, non-significant changes in LDH activity compared to BT values were observed in three training sessions (see Table 1). In addition, in group B, LDH activity increased significantly, although this effect was observed after all three training sessions (Table 2). It should be noted that there were substantial differences between AT values of LDH activity in groups A and B. Statistically significantly higher LDH activity was observed in group B AT I, II, and III in comparison with values in group A (Table 4). In group A, LD1 activity decreased significantly after training series III compared with BT values (Table 1). The adverse effect was observed in group B, where a statistically significant increase in LD1 activity was noted after training series I compared with BT values (Table 2). It should be stressed that significantly higher LD1 activity was noted both BT and AT I as well as AT III in group B in comparison with that in group A (Tables 3, 4). In group A, LD2 activity decreased significantly after training series II and III compared with BT values (see Table 1). In group B, there was a significant increase in LD2 activity after training series I compared with BT value (Table 2). It should be stressed that statistically significantly higher LD2 activity was noted in group B than that in group A BT and AT I and after training series II (Tables 3, 4). In group A, a significant increase in LD3 activity was noted after training series II compared with BT value (Table 1). In group B, a significant increase in LD3 AT I compared with BT value was noted (Table 2). It should be stressed that statistically significantly higher LD3 activity was seen in group B AT I and III than in group A (Table 4). In group A, a significant increase in LD4 activity was seen after training series II compared with BT value (see Table 1). In group B, a significant increase in LD4 activity was seen after all three training series (I, II, and III) compared with BT values (Table 2). It should be emphasized that statistically significantly higher LD4 activity was noted in group B after training series I and III compared with the values in group A (Table 4). In group A, LD5 activity increased significantly after training series II compared with the BT value (Table 1). In group B, there was a statistically significant increase in LD5 activity after training series II and III compared to BT values (Table 2). Higher LD5 activity was seen in group B after training series III than that in group A (Table 4). In group A, the iZO value increased significantly after training series II and III compared with BT values (Table 1). In group B, AT II and III iZO values were also significantly higher compared with BT values (Table 2). It was found that the BT I iZO value was lower and the AT III iZO value was higher in group B than in group A. (Tables 3, 4).

**FIGURE 2:**
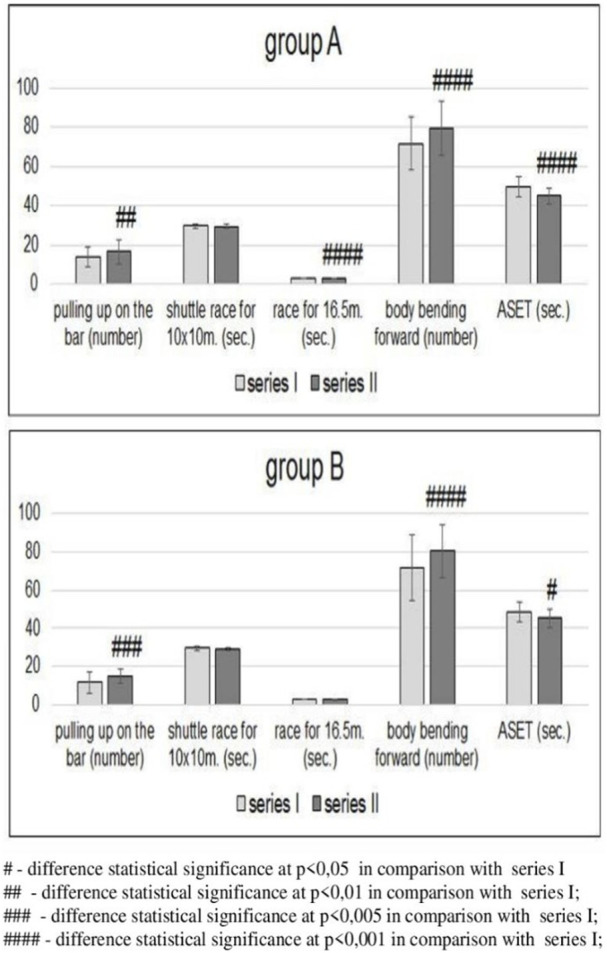
Changes in physical fitness in groups A (n = 41, tested) and B (n = 14, control) before [series I] and after [series II] completion of the education cycle (Wochyński et al., 2010a).

In group A, an analysis of physical fitness showed statistically significant improvement in the series II results in pulling-up on the bar, sprint for 16.5 m, sit-ups, and ASET compared with the results after series I (Figure 2). In group B, an improvement in the results of pulling-up on the bar, sit-ups, and ASET was statistically significant compared with the results of series I. An improvement in the 16.5 m sprint results was insignificant. In both groups, the results of the shuttle sprint for 10 × 10 m proved insignificant in series II compared with the results achieved in series I (Figure 2).

## 4 Discussion

In both groups, the results of the HR measurements showed that the physical exercises involved aerobic metabolic processes but with a higher intensity in group B than in group A. In both groups, changes in CRP and HP levels indicated the absence of an acute phase response after all three training sessions. Our results differ from those of other studies, such as those conducted on marathon runners, in which a statistically significant increase in CRP concentration was observed immediately after the run, and after 24 h protein returned to baseline values (Siegel et al., 2001). During an ultra-endurance race, the greatest hemolysis was observed after 42 km, manifested by a decrease in HP concentration (Yusof, et al., 2007). This is confirmed by earlier studies, in which the authors observed a significant decrease in HP in blood serum after the marathon, with a simultaneous increase in its concentration in urine. The return of HP concentration to the initial values was observed 24 h after the end of the marathon (Sobiech et al., 1992).

The effect of both training regimes on the activities of glycolytic and associated enzymes indicates their role in modulating metabolic processes. Both LDH and aldolase activity were upregulated during training, but their activity was lower in group A during low-intensity training than in group B during the entire exercise cycle (training series I, II and III). This effect likely occurred because the body adapted to the repetitive effort.

In both groups, aldolase activity decreased after training series III compared to BT, which might be due to the fact that under aerobic conditions, ATP production is dominated by oxidative phosphorylation rather than glycolysis. The most probable explanation is that muscle oxidative capacity becomes higher during moderate training, which is also a determinant of endurance capacity. The adaptive process was also pronounced by changes in LDH activity. In group A, after training series I and II, we observed an inconsiderable increase in LDH activity. However, after training series III, we noted a decrease in LDH activity compared to values both before training series III and before whole training program. The more pronounced changes in LDH activity were observed in group B—after all three training series, LDH activity remained elevated. Our findings are in agreement with those reported by studies such as Shin et al. (2016), which found that response to exercise in serum LDH activity depended on training status, as the rise in the enzyme activity was less or even tended to decrease in training but to increase markedly in untrained individuals (Kłapcińska et al., 2001). Moreover, Kobayashi et al. (2005) indicated that intensified aerobic exercise like running may promote elevated LDH activity. Furthermore, it has been suggested that increased training volume and intensity may result in immunological and hormonal changes (Walsh et al., 2011). This might explain higher LDH activity immediately after exercise (Shin et al., 2016). Other authors have also noted that LDH activity increased after a 300 m run (Kłapcińska et al., 2001), after endurance-speed training (Wochyński et al., 1998), and immediately after concentric exercises (Poprzęcki et al., 2004). Lutosławska et al. (2001) showed that LDH activity increased in active men with higher oxygen efficiency after a limit endurance test. In contrast, its activity decreased in individuals of lower efficiency. As they state, the main cause of this phenomenon is a decrease in LD3 and LD4 levels containing a prevalent amount of M subunits (Lutosławska and Sendecki, 1990). Szade et al. (1998) showed a correlation between LD3, LD4, and LD5 after 400 m sprinting. These observations may lead to the conclusion that cadets participating over time have developed (not necessarily consciously) the ability to perform exercises during training series III with the same intensity, but with a lower energy expenditure, than during series I and II. Exercise intensity and its type caused a decrease in LDH activity, probably due to changes in cellular respiration in the muscles. Results from the analysis of *vastus lateralis* muscle biopsies revealed *inter alia* that the maximal oxidative capacity of the skeletal muscle of sedentary individuals, active groups, and athletes increased with the level of physical activity (Zoll et al., 2002). This study indicated that the observed increase is associated with a significant improvement in the coupling between oxidation and phosphorylation in athletic individuals. They also suggest that aerobic capacity and muscle efficiency are not only set by the density of mitochondria but also result from qualitative changes that optimize control of the re-phosphorylation process and the intracellular distribution of high energy phosphates (Zoll et al., 2002). Exercises on SAGI exert an effect on the receptors in the cardiovascular, muscular, and nervous systems. Acceleration in the axis + Gz (head–lower extremities direction) and–Gz (lower extremities–head direction) markedly load the body due to executing exercises in three planes: temporal, fibular, and transverse. Differences in the exercise intensity in the range of aerobic metabolism between groups A and B showed a dissimilar LDH isoenzymatic profile. In group A, a shift of isoenzymes from M to H was seen, whereas the M isozyme functions better in anaerobic environments and the H isozyme functions better in aerobic conditions. This direction of change confirms the marked decrease in LD1 and LD2 after exercise and increase in LD3, LD4, and LD5 after exercise. Such a isoenzymatic profile was found in long-distance, triathlon, and marathon runners (Lutosławska and Sendecki, 1990; Tokinoya et al., 2020). In group B, we observed a significant increase in LDH, LD4, LD5, and iZO activities compared with BT values after training series III. Here, it should be noted that LDH and its isoenzymes activities are statistically significantly higher (except LD2 with insignificant increase) than the values in group A. In well-trained individuals, an increase in AT LDH activity occurs as a result of an increase in LD5 activity (Tokinoya et al., 2020). This is important for understanding changes in the isoenzyme shift caused by intensive physical effort and its type (Shin et al., 2016). To gain in-depth knowledge about isoenzyme shift and body adaptation, the iZO value was assessed. In our previous studies, we demonstrated a higher increase in iZO value in runners for 2000 m than in runners for 4,000 m (Sobiech et al., 2003; Wochyński et al., 2001). We also showed that the higher the iZO value in runners, the shorter the time obtained in both distances at the same type of effort. It is worth emphasizing that in the present study, physical exercise lasted for the same amount of time but the type of training was different. In group A, the iZO AT value tended to decrease from the beginning of the education program (training I) to its completion (training III), while in group B, the tendency was reversed.

LDH isoenzymes are functionally specific. Isoform H favors the production of pyruvate and is inhibited by a high level of pyruvate and its redirection to the citric acid cycle rather than to lactate production (Quistorff and Grunnet, 2011; Gray et al., 2014). During intense training, LDH facilitates glycolytic ATP production by regenerating NAD^+^. With a constant supply of NAD^+^, and as far as acidosis becomes limiting, glycolysis fuels ATP production to support energy demands, which significantly exceed the capacity of oxidative phosphorylation, an energy-efficient but relatively slow process. Increased glycolysis in muscles leads to lactic acid production, which is then transported to the liver, where it is converted back into pyruvate. Pyruvate supports citric acid cycle flux and gluconeogenesis. The entire process is known as the “Cori cycle”.

Body fat mass (FM) along with other marked variables are correlated with overcoming ASET in group A, while in group B there were no such correlations (Wochyński et al., 2010b). Furthermore, regular physical exercise affects the composition of skeletal muscle membrane phospholipids (Mataix et al., 1998; Helge et al., 2001). An increase in oxidative efficiency in well-trained individuals might be explained by changes in the phospholipid membrane structure that affect both its permeability and the efficiency of the ATP synthesis (Brand et al., 1994). In Group B, the training process improved general physical fitness, with the emphasis on endurance during aerobic metabolism of higher intensity. It manifests by an iZO value equal to 0.50 after training series III. iZO equal to 0.50 and higher causes a shift from H to M. The M isoform of LDH remains active at high pyruvic concentration, favoring the production of lactate, while iZO lower than 0.50 shows a shift of isoenzymes from M to H. In group A, iZO values, both before and after training, had a decreasing tendency in all three training sessions of the same intensity. Therefore, the results obtained indicate that the H isoform predominated.

These studies imply that the iZO value is better a diagnostic marker than LDH activity as its value confirms the character of the physical exercise (type of exercise) under the extreme environmental conditions of the pilot’s work. In the case of the tests that assess physical fitness—important as a pilot’s work is associated with extreme environmental conditions—exercises on SAGI exerted a versatile effect on cadets’ motor effectiveness in group A. A higher number of the sport events in series II give significantly better results than those in series I compared with group B. It should be noted that the number of pull-ups on the bar was higher in group A than group B. This might indicate the use of iZO not only for differentiating physical exercise intensity but also its type. Decreased iZO values after training series I, II, and III at the low intensity of exercises are parallel with an emphasis on increased strength endurance. In Group B, an increased iZO value in all three training series (at higher intensity) align with increased endurance. The results of 10 × 10 m and 16.5 m sprints confirmed this.

We want to emphasize that this research will contribute to future experiments, possibly with a larger number of subjects, including a control group, for military aviation candidates. They will also be helpful in training cadets and instructors in dealing with the diagnosis of physiological predispositions to loads on skeletal muscles and physical performance in extreme conditions. The research will also be the basis for modifying the use of special training for pilots and athletes.

## 5 Conclusion

This study’s results show that measuring aldolase and LDH levels are helpful for assessing muscle adaptation to exercise intensity during SAGI training. However, iZO values were a better marker than LDH activity in differentiating the type of physical effort in cadets after SAGI training. We also found that exercises on SAGI did not cause significant changes in the level of acute phase proteins, such as CRP and HP, in the cadets’ bodies. The course of exercises on SAGI showed that iZO values depend on the intensity, type, and adaptation of the physical exercise.

## Data Availability

The original contributions presented in the study are included in the article/supplementary material; further inquiries can be directed to the corresponding author.
